# Beyond breast conservation: is it time to rethink sentinel lymph node biopsy in patients undergoing mastectomy?

**DOI:** 10.1093/bjsopen/zrag132

**Published:** 2026-09-15

**Authors:** Cicero Urban

**Affiliations:** Breast Unit, Hospital Nossa Senhora das Graças and Hospital São Vicente, Curitiba, Paraná, Brazil

Few areas in breast cancer surgery have undergone a more profound transformation than axillary management. Over the past three decades, treatment has evolved from routine axillary dissection to sentinel lymph node biopsy (SLNB), and now towards omission of axillary surgery in selected patients. This evolution has been driven by a better understanding of tumour biology, increasingly effective systemic therapies, and the recognition that less surgery can preserve oncological outcomes while substantially reducing morbidity.

The omission of SLNB in carefully selected patients undergoing breast-conserving surgery (BCS) has become one of the latest milestones in this process. Randomized trials such as SOUND and INSEMA have shown that patients with clinically node-negative, hormone receptor-positive, human epidermal growth factor receptor 2-negative early breast cancer can safely avoid axillary staging without compromising recurrence or survival. Whether these findings can be extended to patients undergoing mastectomy, however, remains an important unanswered question^1,2^.

In this issue of *BJS Open*, Heidiger *et al.*^3^ addresses this clinically relevant gap from a pragmatic perspective. Rather than focusing on oncological outcomes, the authors examine whether occult nodal disease in patients undergoing mastectomy would meaningfully alter recommendations for adjuvant systemic therapy or regional nodal irradiation. This approach reflects contemporary multidisciplinary breast cancer care, in which the value of axillary staging increasingly depends on its impact on treatment decisions rather than anatomical classification alone.

A key finding is the remarkable similarity in axillary disease between patients undergoing mastectomy and those treated with BCS. Although the mastectomy cohort included more multifocal tumours, invasive lobular carcinomas, and less favourable pathological features, these differences did not translate into higher nodal burden or greater indications for chemotherapy, cyclin-dependent kinase 4/6 inhibitors, or regional nodal irradiation^4^. These results challenge the traditional assumption that mastectomy itself identifies a population requiring more extensive axillary staging.

Indeed, contemporary indications for mastectomy often reflect patient preference, genetic predisposition, breast size relative to tumour volume, multifocality, reconstructive considerations, or previous irradiation rather than intrinsically more aggressive disease. Consequently, the type of breast operation should not automatically determine the extent of axillary surgery.

Another important observation is that omission of SLNB would have altered adjuvant treatment recommendations in only a small proportion of patients, regardless of the surgical procedure. This finding reinforces the growing concept that treatment decisions are increasingly driven by biological characteristics rather than nodal anatomy alone. Consistent with this paradigm, lymphovascular invasion emerged as the strongest independent predictor of nodal involvement and additional adjuvant therapy, suggesting that future selection for axillary de-escalation may rely more on biological markers and predictive models than on surgical staging itself.

These findings should nevertheless be interpreted with appropriate caution. This is a retrospective, single-centre study evaluating surrogate endpoints rather than long-term oncological outcomes. In addition, unlike BCS, mastectomy without postmastectomy radiotherapy does not provide incidental irradiation of the low axilla, an important biological difference that continues to justify caution when extrapolating omission strategies between these settings. Definitive evidence will require prospective randomized trials specifically designed for patients undergoing mastectomy.

Despite these limitations, this study contributes meaningfully to the ongoing evolution of axillary management. It redirects attention from the surgical procedure to the biological factors that truly influence prognosis and treatment. If future prospective studies confirm these observations, the distinction between BCS and mastectomy may become progressively less relevant when deciding whether axillary surgery is necessary.

For decades, the trajectory of axillary surgery has consistently moved towards less intervention. The next step may no longer depend on the operation performed on the breast, but on accurately identifying those patients for whom nodal information offers no meaningful therapeutic value. Ultimately, biology—not anatomy—should define the future of axillary management.
